# Prevalence of human immunodeficiency virus 1 infection in the last decade among entry travelers in Yunnan Province, China

**DOI:** 10.1186/s12889-015-1683-8

**Published:** 2015-04-11

**Authors:** Binghui Wang, Yaobo Liang, Yue Feng, Yaping Li, Yajuan Wang, A-Mei Zhang, Zulqarnain Baloch, Li Liu, Weihong Qin, Xueshan Xia

**Affiliations:** Faculty of Environmental Science and Engineering & Faculty of Life Science and Technology, Kunming University of Science and Technology, Kunming, China; Care Center for International Travel Health in Yunnan, Kunming, China

**Keywords:** HIV-1, Prevalence, Infection rate, Cross-border, Border prefecture, Yunnan Province

## Abstract

**Background:**

Yunnan is not only considered the region with the most concerning human immunodeficiency virus (HIV)-1 prevalence, but is also the central hub for the spread of HIV-1 from Southeast Asia to the other provinces of China. Yunnan has the highest proportion of entry travelers who have transmitted HIV from neighbored Southeast Asian countries to mainland of China.

**Methods:**

Between 2003 and 2012, we recruited 280,961 entry travelers at land ports located in 7 bordering prefectures respectively in the Yunnan Province for HIV-1 screening. Based on the detection of HIV-1 antibody, the HIV-1 infection rate was determined.

**Results:**

Among the recruited entry travelers, 2380 were determined HIV-1 positive with infection rate of 0.85%. Travelers entering the Dehong port had the highest HIV-1 infection rate (5.12%), followed by those entering Baoshan (0.88%), Lincang (0.83%), and Honghe (0.71%). For all HIV-1 positive cases, travelers aged 21–30 and 31–40 were the most commonly infected individuals, accounting for 38.45% and 37.77% of all cases, respectively. The most common occupation of the infected population was driver (42.38%), and the proportion of industrials had increased yearly. Based on the reported risk factors, sexual transmission was the main HIV-1 infection route (77.11%) of this population.

**Conclusions:**

We have clarified the rate of HIV-1 infection among this bridge population. The characteristics of HIV-1 positive population and high geographical heterogeneity have provided the necessary epidemiological data for monitoring the HIV-1 epidemic among cross-border travelers in Yunnan and to further understand the cross-border spreading of the HIV-1 infection.

**Electronic supplementary material:**

The online version of this article (doi:10.1186/s12889-015-1683-8) contains supplementary material, which is available to authorized users.

## Background

Globally, human immunodeficiency virus (HIV)-1 infection and mortality rates have been declining because of the highly active antiretroviral therapy and effective prevention measures. However, an estimated 35.3 million people (range, 32.2–38.8 million people) were still living with HIV-1 in 2012 [1]. Southeast Asia was once considered the center of the HIV epidemic worldwide, with approximately 3.5 million HIV-1 infected individuals living in this area [2,3]. Various HIV-1 subtypes/recombinants have been reported to be co-circuiting, and novel circulating recombinant forms (CRFs) have been frequently identified in Southeast Asia in recent years [4-7]. Although the HIV infection rate is still low, there are still >434,000 infected people living in China because it has the largest population in the world. Between January 2013 and September 2013, more than 70,000 individuals were newly infected with HIV-1 [8].

The Yunnan Province is located in Southwest China and borders the Southeast Asian countries. The first local HIV-1 infection case in the Yunnan Province was identified in 1989 in the Dehong Prefecture. Subsequently, Yunnan was considered the epicenter of HIV-1 prevalence in China and the hotspot where HIV-1 recombinants developed [9-11]. By the end of 2013, >83,000 people were living with HIV-1 in the Yunnan Province. With the changes in major HIV-1 risk behaviors, the heterosexual spreading of HIV-1 is gradually increasing as the main transmission route, compared to syringe/needle sharing, in this region [12,13]. HIV-1 genetic diversity has been reported to be more complex in Yunnan than in any other region of China [14]. Because of its special geographic location and frequent personnel exchange with the Southeast Asian countries, Yunnan is not only considered a region with the most concerning HIV prevalence, but it is also the central hub for the spread of HIV-1 from Southeast Asia to the other provinces of China [10,11,15].

Because of its deepening reform and open policy, the number of cross-border travelers in China was as high as 454 million in 2013. Yunnan has the largest population of cross-border travelers after Xinjiang Autonomous Region, with most of these travelers originating from Southeast Asia. As the major community of international business and trade, cross-border travelers and their activities often lead to the spread of infectious diseases. A complex distribution of some blood-borne pathogens in Yunnan may be associated with the exchange of this population [16]. Although HIV infection among cross-border travelers has been reported [17,18], the HIV-1 infection rate and other epidemic characteristics of this bridge population in the Yunnan Province are still unclear.

Thus, it is necessary to monitor the prevalence of HIV-1 among cross-border travelers, particularly the entry personals. Moreover, an investigation of temporal changes in HIV-1 infection and the related risk factors could have significant implications on HIV-1 prevention. Therefore, to clarify the HIV epidemic characteristics among cross-border travelers, HIV-1 screening was conducted on a large scale among cross-border travelers who entered Yunnan through the major land ports located in border prefectures in last decade and a further analysis of the risk factors was performed in the current study.

## Methods

### Study population and data collection

Between January 2003 and December 2012, there were about 16 million entry travelers on average through the selected ports in Yunnan province annually. We recruited 280,961 entry travelers (3.63‰) across the border in Yunnan Province, China. The local staffs of land ports were required to invite one for routine physical examinations every five-hundred travelers. The entry travelers from the same prefecture are classified as a group in spite they entering at different land ports. Therefore, 13 land ports was covered including 3 in Dehong, 1 in Baoshan, 3 in Lincang, 2 in Puer, 2 in Xishuangbanna, 1in Honghe, 1 in Wenshan. Routine physical examinations including complete blood counting, blood chemistries and the detection of infectious pathogens (Hepatitis B virus, Hepatitis C virus, Syphilis, Malaria, Dengue virus and Typhoid fever virus) were conducted. Data on the social-demographic characteristics and risk behaviors for HIV infection were obtained from the enrolled participants via face-to-face interviewing with trained staff by administering a structured questionnaire (a blank example of questionnaire was given to illustrate in Additional file 1).

### Detecting the human immunodeficiency virus-1 infection

Blood samples were collected from the participants. The isolated serum was submitted for preliminary HIV antibody detection using dipstick screening (Alere Determine HIV-1/2; Alere Medical Co., Ltd., Tokyo, Japan). A second screening was performed using the Genscreen ULTRA HIV Ag-Ab kit (Beijing WANTAI Biological Pharmacy Enterprise Co., Ltd., Beijing, China) at the local HIV testing outreach laboratory. Suspected HIV-1 positive samples were transported to the central laboratory in Kunming, China, within 12 hours for evaluation using enzyme-linked immunosorbent assay (ELISA; Beijing BGI-GBI Biotech Co., Ltd., Beijing, China). Finally, positivity was confirmed using Western blot analysis (WB; MP Diagnostics Co., Ltd., Singapore).

### Statistical analysis

All recruited individuals were categorized according to sampling year, ports’ location, age and occupation. The statistical analysis was conducted using the SPSS, version 12.0 software package (SPSS Inc., Chicago, IL, USA). Characteristics were compared between the groups using *χ*^2^ tests, and results with P values of <0.05 were considered statistically significant.

### Ethics statement

All the participants were informed about the study before participation, and written informed consent was obtained at sample collecting. The study design was approved by the Kunming University of Science and Technology’s Institutional Ethics Committee.

## Results

### Demographic characteristics and infection rates

Of the 23 entry ports in the Yunnan Province, 19 land ports communicate via rail or road with Myanmar, Laos and Vietnam. Between 2002 and 2012, we recruited 280,961 entry travelers who entering at 13 land ports located 7 bordering prefectures respectively in Yunnan Province for HIV-1 infection screening. These prefectures are lined along the border of Yunnan Province from west to east. The annual recruited population (range, 31,749–45,495) did not differ significantly between the sampling years. Of the recruited participants, 22,699 were from Dehong, 17,074 from Baoshan, 58,195 from Lincang, 51,698 from Puer, 91,432 from Xishuangbanna, 22,799 from Honghe, and 17,064 from Wenshan.

**Figure 1:**
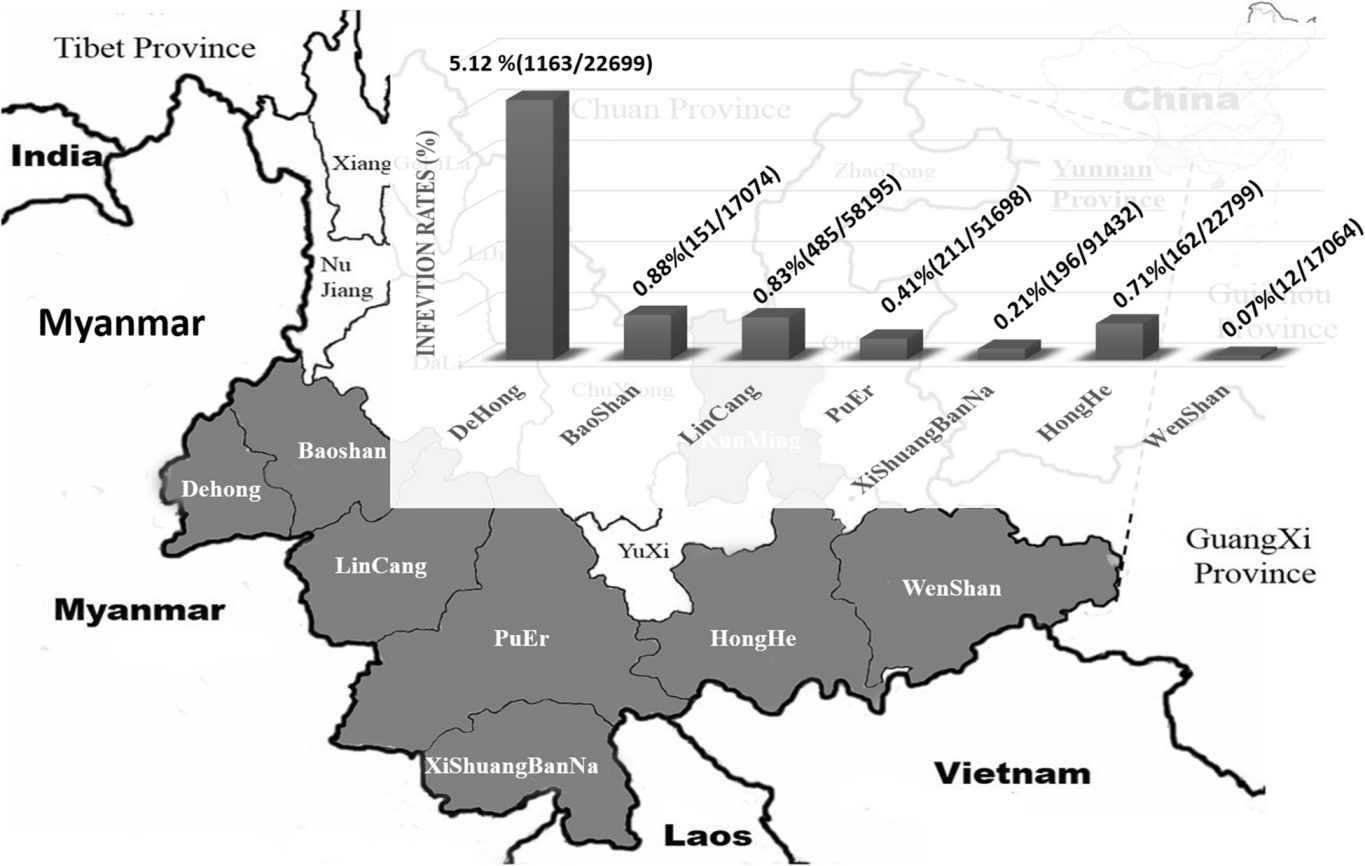
The geographical position of 7 important land border ports, and the HIV-1 infection rates of each port. The asterisk in the map shows the geographical position of these seven border prefectures in this study.

Based on ELISA and WB results, 2,380 individuals were detected to be infected with HIV-1, resulting in an infection rate of 0.85%. The HIV-1 infection rates differed significantly among the 7 border prefectures. Travelers entering at Dehong, the main ports in the southwest of the Yunnan Province bordering Myanmar, had the highest HIV-1 infection rate (5.12%, 1,163/22,699), which was 72 times higher than that among travelers entering at Wenshan (0.07%, 12/17,064), which was the lowest (Figure 1). The second highest infection rates (>0.50%) were found among travelers entering at Baoshan (0.88%, 151/17,074), Lincang (0.83%, 485/58,195), and Honghe (0.71%, 162/22,799). The remaining prefectures, Puer (0.41%, 211/51,698) and Xishuangbanna (0.21%, 196/91,432) had lower infection rates.

### Characteristics of the human immunodeficiency virus-1 infected travelers

Of 2,380 HIV-1 infected cross-border travelers entering at these border prefectures, 1,619 (68.02%) were male and 761 (31.98%) were female, with a male-to-female ratio of 1.88:1 and an age range of 14–78 years (median, 32.8 years). Those aged 21–30 years and 31–40 years accounted for 38.45% and 37.77% of the infected population, respectively. More than 5 occupations were prominent among the infected individuals. Drivers, merchants, farmers, industrials, and servicers accounted for 39.92%, 15.30%, 17.43%, 10.52%, and 7.53% of all infected individuals, respectively (Table 1). Of the 1,961 infected individuals for whom data on nationality were available, more than half were Burmese (1,153 cases, 58.80%), 713 (36.36%) were Chinese, 81 (4.13%) were Vietnamese, and the remaining 14 (7.14%) were from other countries. The route of transmission was clearly expressed by 319 HIV-1 infected individuals. According to the provided information, most of individuals (77.11%) were infected with HIV-1 through sex, and 18.19% were infected via intravenous drug use (syringe/needle sharing).

**Table 1:** **The characteristics of HIV-1 infected individuals**

**Characteristic**		**N** ^**a**^	**N**	**%**
Sex		2380		
	Male		1619	68.02
	Female		761	31.98
Age, year		2346		
	≤20		149	6.35
	21-30		902	38.45
	31-40		886	37.77
	41-50		293	12.49
	51-60		92	3.92
	>60		24	1.02
Occupation		2347		
	Driver		937	39.92
	Merchants		359	15.30
	Farmer		409	17.43
	Industrials		247	10.52
	Services		186	7.53
	Others		209	8.90
Education, years		832		
	<6		109	13.10
	6		281	33.78
	9		308	37.03
	12		101	12.14
	>12		33	3.97
Route of transmission		319		
	Sex		246	77.11
	IDU		58	18.19
	Iatrogenic infection		15	4.70
Nationality		1961		
	Burmese		1153	58.80
	Chinese		713	36.36
	Vietnamese		81	4.13
	Others		14	0.71

^a^The number of population in each category may not add up to 2380 because of the obtained inconsistent information.

### Annual human immunodeficiency virus-1 infection rates among entry travelers in Yunnan

**Figure 2:**
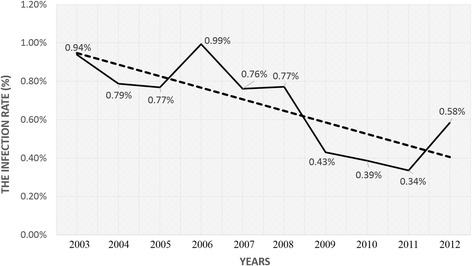
Trend of Yunnan cross-border travelers’ HIV-1 infection rates in the past decade.

**Figure 3:**
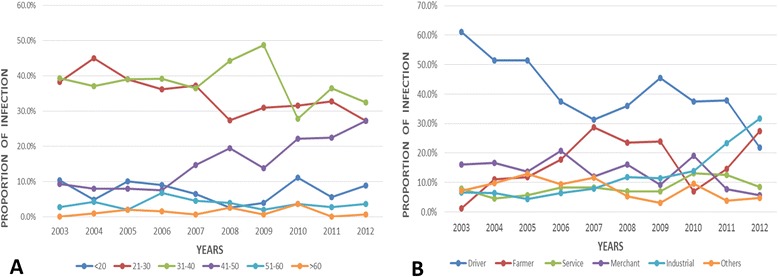
The age group distribution **(A)** and Career distribution **(B)** of infected individuals in each year in Yunnan province.

According to the results of HIV-1 screening, the HIV-1 infection rate showed a decreasing trend overall (Figure 2), although the infection rate of 2006 seemed to be is abnormal. Before 2009, the infection rates were between 0.70%–0.90%. Subsequently, the infection rate decreased to <0.50%, although the rate in 2012 was higher (0.58%). There were no significant differences between annual data on age and occupation over these 10 years. Individuals aged 21–30 years and 31–40 years were the most commonly infected. However, the proportion of those aged 40–50 years showed an increasing trend, from 9.35% in 2003 to 27.21% in 2012 (Figure 3A). The proportion of industrials increased annually, whereas the proportion of drivers decreased (Figure 3B). There were no obvious trends in the proportions of infected individuals with other occupations (i.e., farmers, servicers, merchants, and others).

## Discussion

According to the released travel data from the Tourism Bureau of Yunnan Province, the total number of entry travelers rapidly increased from 1.01 million in 2000 to 4.58 million in 2012. The nearly 5 times increase in foreign entry travelers in the past decades reflects the more frequent exchanges of international personnel in the Yunnan Province. Previous studies on the HIV-1 epidemic in this region have mainly focused on injection drug users (IDUs) [19,20], female sex industrials (FSWs) [12,21,22], sexually transmitted disease (STD) clinic attendees, and men who have sex with men (MSM) [23]. This is the first study to determine the HIV-1 infection rate among cross-border travelers [12] and their characteristics over such a long time span (from 2003 to 2012) and with a large-scale population (280,961 cases) in Yunnan, China.

The HIV-1 infection rate among entry travelers (0.85%) is lower than that among high-risk populations (FSWs, 2.20%; IDUs, 40%; and MSM, 13.2%) [23], but significantly higher than that among the general population in the Dehong Prefecture (5.124% vs. 1.3%; P < 0.01) [24]. People who have transmitted HIV from a high-risk population to the general population are defined as bridge population [13]. Cross-border travelers with an HIV-1 infection may cause local HIV-1 transmission and are defined as a bridge population [13]. Most of the entry travelers recruited in this study were from Southeast Asia, which is considered one of the epicenters of the HIV epidemic worldwide [2,3]. Undoubtedly, the existence of the bridge population may accelerate the spread of HIV-1 into China from the Southeast Asian countries.

Overall, we found that the HIV-1 infection rate in this population showed a decreasing tendency over the 10 years, verifying that the HIV/acquired immune deficiency syndrome (AIDS) prevention and control work, such as the promotion of condoms in Southeast Asian countries and implementation of the Asian regional AIDS project, has received extensive domestic and international attention. These programs also have a good effect in Yunnan and the neighboring Southeast Asian countries.

In view of the age-specific HIV-1 prevalence, the major age group of HIV-1 infected travelers was 21–40 years (76.20%). The proportion of individuals aged 21–30 years increased annually. Although Jia et al. reported a decreasing tendency in the HIV-1 infection rate among those aged 31–40 years in the general population of Yunnan in 2010 [12], this was not observed in the current study population. This difference may be caused by the different study subjects; they focused on the general population. Drivers was accounted for the major occupation of the HIV-1 infected entry travelers due to the occupation limitations of entry personals in this study. Therefore, HIV monitoring on this sub-population is particularly necessary. Although HIV-1 infection was suspected to be inversely related to the level of education, individuals who completed 6 and 9 years of education comprised the commonly infected groups in this study. Among 319 individuals who clearly indicated the route of infection, sexual contact accounted for 77.11% of the HIV-1 transmission routes. This is consistent with the fact that sexual transmission has increased and has gradually become the main route of transmission compared to syringe/needle sharing among IDUs [12,13].

Dehong was once considered one of the most harmful prefectures of mainland China in terms of HIV/AIDS prevalence, with 17,590 cumulative reported HIV/AIDS cases at the end of 2010, representing 6.4% of the total reported HIV/AIDS cases in China [24,25]. In this study, the HIV-1 infection rate among travelers entering the Dehong port was significantly higher than that among travelers entering other ports (5.12% vs. 0.41%, P < 0.10). The Dehong Prefecture borders Myanmar where HIV infection is a serious concern because of drug production and frequent drug use [26,27]. The population of cross-border travelers at the Dehong port is larger than that of at all other ports of the Yunnan Province. There were >29.35 million cross-border activities via the Dehong port in 2013. The serious HIV epidemic in Dehong may be attributable to this large cross-border population with a high HIV infection rate. This finding further suggests that entry travelers may influence HIV-1 prevalence in the local general population. High HIV-1 infection rates were also observed among entry travelers at the ports of Baoshan and Lincang, which also bordering Myanmar and are neighbored with Dehong.

The epidemic of a variety of blood-borne viruses was proven to be influenced by the transmitted strains from the Southeast Asian countries [28-30]. Cross-border travelers are the bridge population between China and Southeast Asian countries. Study of the HIV-1 infection rate in this bridge population revealed its decreasing tendency over the past decade and a high geographical heterogeneity, which provide the necessary epidemiological data for monitoring the HIV-1 epidemic among entry travelers in Yunnan and to further understand cross-border HIV-1 transmission.

Although the HIV-1 prevalence among these recruited 280,961 cross-border travelers who entered at major land ports in last decades were described, there are still several limitations need to be complemented. Firstly, there are 8 border prefectures in Yunnan; because of the lesser crossing border travelers and the irregular information, Nujiang Prefecture was excluded from this study. Secondly, the characteristics of HIV-1 prevalence among this recruited population was needed to be further confirmed due to the lack of detailed information for unwilling of some participants. Nevertheless, the monitoring on HIV-1 prevalence among this bridge population in last decades is vital for understanding on the HIV-1 cross-bordering transmission.

## Conclusion

In conclusion, a high infection rate and decreasing tendency were the main features of the HIV-1 epidemic in Yunnan’s cross-border traveler population in the last decade. We have described in detail the infection rate distribution among border prefectures in Yunnan. Our study findings suggest that the HIV-1 epidemic in this bridge population warrants close attention, and the cross-border movement of this population will have far-reaching implications on the global transmission of HIV.

## Additional file

Additional file 1:
**The registration form of HIV-1 positive case.**
