# Supplementary material for: Prevalence of human immunodeficiency virus 1 infection in the last decade among entry travelers in Yunnan Province, China
Source: BMC Public Health. 2015 Apr 11;15:362. doi: 10.1186/s12889-015-1683-8 (PMC4415269; doi:10.1186/s12889-015-1683-8)
Supplement: Additional file 1: — The registration form of HIV-1 positive case. [file 12889_2015_1683_MOESM1_ESM.pdf]

The Registration Form of HIV - 1 Positive Case

NO.： ________________________ Name: ____________________

Detection Units:_________________ Detection Date: ______________

ID Number:_____________________ Gender:_____________________

Career:________________________ Date of birth:________________

Marital Status: ________________

Residence Location: ____

Work Units:____

Educational Degree:

<6 years ☐ 6 years ☐

9 years ☐ 12 years ☐

>12 years ☐

Whether have tested positive for HIV – 1 before?

Yes ☐ When? ________________

No ☐

Which one do you think is your most likely route of infection?

Unprotected sex ☐

Intravenous drug use ☐

Organ transplantation or extracorporeal blood transfusion ☐

Don't know ☐

Others:________________

What is your purpose in applying for a visa?

____

Note: this form is for personal interview only.
